# MRI assessment of diastolic dysfunction in comparison to transthoracic echocardiography

**DOI:** 10.1186/1532-429X-14-S1-P304

**Published:** 2012-02-01

**Authors:** Sebastian Buss, Birgit Krautz, Florian Andre, Evangelos Giannitsis

**Affiliations:** 1 Department of Cardiology, University of Heidelberg, Heidelberg, Germany

## Summary

We could show that PC-CMR analysis of DD is clinically feasible and showed excellent agreement with the widely accepted EC parameters for classification of DD. PC-CMR could offer the potential of a practically and reasonably time-consuming approach for the clinically important assessment of DD with CMR.

## Background

Over the last decade the interest in diastolic dysfunction has grown, due to the fact that at least half of all cases of newly diagnosed heart failure patients present with preserved ejection fraction. One of the most important tasks of cardiovascular imaging is the objective assessment of left ventricular (LV) systolic and diastolic function, which is routinely performed with standard trans-thoracic echocardiography (EC). In EC, classification of diastolic dysfunction (DD) is widely accepted and mainly assessed using three criteria: mitral blood flow (MBF, E-A-curve), lateral wall velocity (LWV, S-E-A-curve) and pulmonary vein flow (PVF, S-D-AR-curve). With these three characteristic flow and velocity patterns and their ratios E/A, E/E and S/D, regular diastolic function can be clearly distinguished. Cardiovascular magnetic resonance (CMR) has excellent capabilities to assess blood flow and myocardial tissue motion using phase contrast (PC-CMR) imaging. We sought to compare the feasibility of PC-CMR with echocardiographic doppler imaging for the assessment of DD using the echocardiographic flow and velocity approach for DD-classification.

## Methods

After acquisition of regular short axis cine SSFP volumetry and 2-3-4-chamber views in 38 patients with various cardiovascular diseases, we performed single-slice short-axis PC-CMR (60phases,velocity-encoding=100cm/s) similarly to typical EC locations at the tip of mitral leaflets in diastole on a 1.5T whole body MRI system (Philips Achieva) to generate mitral E-and A-waves, lateral S`-E`-A-velocities, E/A- and E/E`-ratios. PC-CMR for PVF was planned orthogonally to the cine 4-chamber plane 1cm distal from pulmonary vein inflow into the left atrium. Directly after MRI, EC was performed to generate complementary data for MBF, LWV and PVF.

## Results

EC and PC-CMR could be performed in all patients, whereas EC PVF could not be assessed in 8 patients due to reduced flow signals. Correlation with mitral E and A velocities was good (r=0.72 and 0.74, p<0.001) as well as their correlation (r=0.76, p<0.001). The correlation for E/E’, as one of the most clinically relevant parameters, was high with r=0.79 (p<0.001). 36/38 patients (95%) were categorized the same by both CMR and EC, whereas in 2 cases PC-CMR mis-diagnosed them as normal. Mean scan-time for MBF, LWV and PVF was 5.20±1.31 minutes, mean analysis time was 4.10±1.31 min.

## Conclusions

We could show that PC-CMR analysis of DD is clinically feasible and showed excellent agreement with the widely accepted EC parameters for classification of DD. PC-CMR could offer the potential of a practically and reasonably time-consuming approach for the clinically important assessment of DD with CMR.

## Funding

No funding.

